# Analysis of Plant-Bacteria Interactions in Their Native Habitat: Bacterial Communities Associated with Wild Tobacco Are Independent of Endogenous Jasmonic Acid Levels and Developmental Stages

**DOI:** 10.1371/journal.pone.0094710

**Published:** 2014-04-11

**Authors:** Rakesh Santhanam, Karin Groten, Dorothea G. Meldau, Ian T. Baldwin

**Affiliations:** Department of Molecular Ecology, Max-Planck-Institute for Chemical Ecology, Jena, Germany; Wageningen University and Research Centre, The Netherlands

## Abstract

Jasmonic acid (JA) mediates defense responses against herbivores and necrotrophic pathogens but does it influence the recruitment of bacterial communities in the field? We conducted field and laboratory experiments with transformed *Nicotiana attenuata* plants deficient in jasmonate biosynthesis (ir*AOC*) and empty vector controls (EV) to answer this question. Using both culture-dependent and independent techniques, we characterized root and leaf-associated bacterial communities over five developmental stages, from rosette through flowering of plants grown in their natural habitat. Based on the pyrosequencing results, alpha and beta diversity did not differ among EV and ir*AOC* plants or over ontogeny, but some genera were more abundant in one of the genotypes. Furthermore, bacterial communities were significantly different among leaves and roots. Taxa isolated only from one or both plant genotypes and hence classified as ‘specialists’ and ‘generalists’ were used in laboratory tests to further evaluate the patterns observed from the field. The putative specialist taxa did not preferentially colonize the jasmonate-deficient genotype, or alter the plant's elicited phytohormone signaling. We conclude that in *N. attenuata*, JA signaling does not have a major effect on structuring the bacterial communities and infer that colonization of plant tissues is mainly shaped by the local soil community in which the plant grows.

## Introduction

Plants are inhabited by diverse bacterial communities which live in and on the plant's tissues. Bacteria which have been clearly shown to colonize tissues internally are termed “endophytes”; though in literature the term has also been extensively applied to bacteria or DNA extracted from surface-sterilized plant tissue [1], [2]. In the introduction we will use the term for both definitions, but are aware that after surface-sterilization not all of the isolated bacteria or DNA might be strictly derived from internal tissues, as some bacteria may have survived the sterilization treatment, and that tissue-associated bacteria would be a more appropriate term for our own findings.

Endophytes or particular bacterial isolates can either have beneficial or detrimental effects on their hosts [1], [2]; some are seed borne [3], but most bacteria are recruited from the surrounding soil during germination [4]–[6]. Thus, plants grown in different soils harbor highly diverse bacterial communities [7], and increasingly evidence for plant-soil feedbacks [8], [9], likely mediated by root exudates, such as amino acids, sugars, fatty acids and organic acids, are being shown to affect soil bacterial communities. Many studies have revealed that plants only recruit a selection of the bacteria present in their immediate surroundings, and bacteria from the phyla Actinobacteria, Bacteroidetes, Firmicutes, and Proteobacteria are found most frequently in roots [6], [10]–[12]. However, further research is needed to determine how the soil microbiome and plant-microbe feedbacks influence the populations of bacterial communities.

Bacterial communities are highly diverse among the different tissues, and they are found in seeds, roots, leaves, stems, tubers, ovules and fruits [2], [13]. For some plant species, roots harbor a greater number of bacterial taxa compared to stems and leaves and the communities differ in the different tissues [14], [15]. The communities also differ among plant species [16] and genotypes [17], [18] and change seasonally and developmentally [19], [20]. Overall, it remains unknown if and how biotic or abiotic stresses and ontogeny affects the composition of the endophyte or tissue-associated bacterial community [21], [22].

The phytohormones, salicyclic acid (SA), jasmonic acid (JA) and ethylene (ET) regulate responses to biotic and abiotic stresses [23]–[25] and play central roles in coordinating various aspects of developmental processes throughout the life cycle of plants, including flower morphogenesis, fruit formation or ripening, seed germination and root elongation [26], [27], but they also play a major role in mediating defense responses against herbivores and pathogens [28], [29]. Several plant-growth promoting (PGP) bacteria have been shown to enhance a plant's resistance against biotrophic and necrotrophic pathogens by increasing SA and JA levels, respectively [30]–[33]. These studies have mostly focused on effects on the pathogen community; far less is known about how these phytohormones influence the bacterial communities. Only two studies have examined this question with *Arabidopsis* plants and with contradictory results [34], [35].

In coyote tobacco (*Nicotiana attenuata*), it was previously shown that the root-associated bacterial community is influenced by ET perception and production [5]. However, the influence of JA and JA-inducible defenses on bacterial communities remains an open question. In this species, the herbivory-induced accumulation of ET, JA and JA-isoleucine (JA-Ile) is influenced by ontogeny; in particular the onset of flowering strongly reduces the inducibility of these three signaling molecules [36]. A systematic analysis of plant ontogeny and JA effects on the tissue-associated bacterial community composition is clearly needed for this native plant which has become a model for the study of ecological interactions.

In this study, *N. attenuata* (Solanaceae), a tobacco native to the Great Basin Desert that germinates after fires from long-lived seed banks to form monocultures in the nitrogen-rich soils of the post-fire habitat [43], was used as a model plant. Its defense reactions against attack from specialist (for example, *Manduca sexta*) and generalist herbivores have been extensively studied [44]. Natural or simulated attack by *M. sexta* larvae elicits strong JA signaling which in turn leads to the production of various defense responses [45]. This JA signaling can be silenced by knocking down the expression of a key enzymatic step in JA biosynthesis, allene oxide cyclase (AOC) which converts 13S-OOH-18:3 to 12-oxo-phytodienoic acid (OPDA), and is encoded by a single gene. OPDA is subsequently transformed into JA by reduction and three cycles of β-oxidation [46], [47]. To date, the influence of JA signaling on the bacterial communities have not been thoroughly examined and plants impaired in JA biosynthesis provide an important tool to reveal the role of JA in shaping bacterial communities.

In this study we used plants differing in endogenous JA levels grown in their native habitat and tested the hypothesis that variation in JA signaling defense pathways, a trait that is known to be variable amongst different genotypes found within native *N. attenuata* populations [47] and to change over plant development [36] affects the root- and leaf-associated bacterial community composition and diversity. We planted two isogenic size-matched cohorts of *N. attenuata* plants with normal (EV) and impaired JA-biosynthesis (ir*AOC*) in their natural environment in Utah, USA, and harvested roots and leaves at 5 different developmental stages from rosette through flowering during the 2012 field season. Bacterial communities were retrieved by a combination of culture independent (pyrosequencing) and dependent approaches [37], [38], [40]–[42]. Statistical analyses and diversity indices were employed to assess the effects of JA signaling on the bacterial diversity in roots and leaves. In order to, further explore if the two genotypes specifically recruit particular bacterial isolates under *in-vitro* conditions. We investigated the effect of JA signaling on the bacterial colonization, but also if inoculation influenced the levels of JA, SA and ET after elicitation of EV plants by treating fresh puncture wounds with *M. sexta* oral secretion (OS_MS_).

## Materials and Methods

### Plant material for field and glasshouse studies and sample collection in the field

For the field experiment, previously characterized, homozygous *Nicotiana attenuata* Torr. ex Watson empty vector plants (EV, A-03-9-1-1,[48]) and an isogenic transgenic line impaired in JA biosynthesis (ir*AOC*, A-07-457-1, [47]) were used. EV and ir*AOC* plants were germinated on Gamborg B5 as described in Krügel *et al*. [49], transferred to individual Jiffy pots and planted in size-matched pairs in a field plot located at Lytle Ranch Preserve, Great Basin Desert, Utah [50]. Plants were harvested at different developmental stages and rosette diameter and stalk length for each stage are shown in (Table S1 in File S1). At harvest plants were separated into roots and leaves and washed in tap water to remove the soil particles attached to the roots and transported to the laboratory on ice.

### Isolation of culturable root- and leaf-associated bacteria

Two days after excavation from the field, bacterial isolation was carried out as described in [5]. Roots and leaves were surface-sterilized and a fraction stored at −80°C for pyrosequencing [51], while the remaining tissue was aseptically sectioned into smaller fragments and distributed onto three different isolation media: tap water-yeast extract agar (TWYE [52]), *Streptomycetes* isolation media (SIM [53]) and glucose-yeast extract agar (GYE [54]). Plates were incubated at 28°C for 4 d. After incubation, colonies were picked from plates, sub-cultured and stored in 50% glycerol solution at −80°C. The total number of bacterial isolates recovered from the respective media were 116 from EV roots (GYE-40, TWYE-42, SIM-34), 89 from ir*AOC* roots (GYE-32, TWYE-32, SIM-25), 107 from EV leaves (GYE-38, TWYE-41, SIM-28) and102 from ir*AOC* leaves (GYE-37, TWYE-35, SIM-30). The surface sterilization procedure efficacy was assessed by plating aliquots of the sterile distilled water used in the final rinse onto nutrient agar medium (Sigma, Steinheim, Germany) and incubated as described above. We did not observe any bacterial colonies on control plates.

### Bacterial DNA extraction and 16S rRNA gene sequencing and identification

Genomic DNA was extracted from bacterial isolates and16S rRNA, PCR amplifications were performed according to Kim and Goodfellow [55] with minor modifications. Amplification of 16S rRNA gene was performed in a 20 µL final volume of ReadymixTaq PCR reaction mix (SigmaAldrich) containing 2 µL of template DNA, 50 µM of primer 27F (5′-AGAGTTTGATCCTGGCTCAG- 3′) and 1492R (5′- GGTTACCTTGTTACGACTT- 3′, [56]. A negative control PCR mixture with sterile water was included in all PCR experiments. PCR products were purified using the QIAquick™ Gel Extraction Kit (QIAGEN, Hilden, Germany) following the manufacturer's manual. Direct sequencing using the primer 783R (5′- CTACCAGGGTAT C TAATCCTG -3′) was conducted with Big Dye Mix (Applied Biosystems, Foster City, CA, USA), and purification of the sequencing reactions was performed using the Nucleo-SEQ Kit (Macherey-Nagel, Düren, Germany). Analysis of all sequences was carried out in EzTaxon server (http://eztaxon-e.ezbiocloud.net/, [57])

### Plant DNA extraction, sample pooling and bacterial tag-encoded FLX amplicon pyrosequencing (bTEFAP), and 16S rRNA analysis

Total genomic DNA was extracted from all surface-sterilized root and leaf tissues using FastDNA™ Spin kit for soil (MP biomedicals). DNA of biological replicates at the respective developmental stages of the EV and ir*AOC* genotypes (n = 3–5) were pooled into one DNA sample (total number of pooled samples  = 20): the concentration was determined by NanoDrop spectrophotometer, and diluted to a working concentration of 30 ng/µL before combining equal volumes. To evaluate the utility of 3 different primers, rosette-stage leaf and root samples were used. Bacterial 16S rRNA genes variable regions from V4–V9 were amplified by the following 3 primers sets: 515F-806R: GTGCCAGCMGCCGCGGTAA - GGACTACVSGGGTATCTAAT [58]; 799F-1394R: ACCMGGATTAGATACCCKG- ACGGGCGGTGTGRTC, [59] and 939F-1394R: TTGACGGGGGCCCGCAC- ACGGGCGGTGTGRTC. The 799F-1394R primers were used in all subsequent analyses. Pyrosequencing bTEFAP was performed by Research & Testing Laboratories, Lubbock, TX, USA (RTL, www.researchandtesting.com).

### Pyrosequencing 16S rRNA gene sequence analysis

The QIIME software package was used to analyse the high-quality reads using default parameters for each step [60]. Briefly, sequences were eliminated if the average quality scored <25, lengths were shorter than 200 bp, excess of 6 bases homopolymer runs, primer mismatch and ambiguous bases. USEARCH series of scripts were used to remove the chimer and noisy sequences followed by clustering of OTUs picking with 97% cut-offs [61]. Most abundant sequences were taken as representative sequence for each clusters and aligned to the Greengenes database [62] using PyNast algorithm with minimum percent identity at 80% [63]. FastTree was used to build the phylogenetic tree [64] and taxonomy was assigned using RDP classifier with a minimum support threshold at 80% [65]. OTUs with the same taxonomy at class and genera-level were pooled for description of community.

### Statistical analysis

Primer E software v.6 [66] and QIIME software package were used for all statistical analyses. All samples were rarefied and OTUs present in ≤2 samples were not considered for further analysis. Alpha-diversity was determined by calculating the Shannon diversity index [67], Margalef's index [68], and Pielou's evenness index [69] based on OTUs 97% identity. The mean of 10 permutations of richness, evenness and diversity were used for an ANOVA analysis to compare ‘genotype & tissues’ (EVL, ir*AOC*L, EVR, ir*AOC*R). QIIME script OTU significance test (ANOVA) was applied to find out whether OTUs based on 97% identity are significantly associated with a specific sample type. The Uni-Frac distance metric was calculated as a measure of bacterial community similarity [70] for roots and leaves of each plant developmental stage. Its values range between 0 to1, and samples with a value of 1 have entirely different bacterial communities, while 0 indicates bacterial communities are identical among two samples. The Uni-Frac distance metric was also the basis for non-parametric analysis of similarities (ANOSIM) among samples [71] and non-parametric multidimensional scaling (MDS) to visualize the similarity of bacterial communities among genotype roots and leaves at the different developmental stages [72]. Alpha and beta diversity, Uni-Frac distance metric and Uni-Frac beta significance test were calculated based on 97% sequence identity. ANOVA followed by Fisher's PLSD test was used to compare means of CFU g^−1^ fresh mass (F-mass) of fresh roots or leaves, root length, plant biomass, leaf-surface area, and phytohormone levels (JA, SA, ET) using Infostat 2010 [73].

### *In vitro* bacterial re-colonization assays

In order to examine whether JA influences the colonization pattern of bacteria isolated from the field, we selected ten bacterial strains exclusively isolated from either of the plant genotypes (EV or ir*AOC*, called ‘putative specialist’) and seven bacterial strains isolated from both genotypes, dubbed ‘putative generalists’, and *Bacillus sp* B55 from a previous study as positive control [74], for specific colonization assays. Roots of 7-day-old seedlings were dipped into 1 mL of bacterial suspensions for 1 min (OD_600_  = 1) and transferred to a Magenta™ vessel box (W×L×H; 77 mm ×77 mm ×97 mm, Sigma, GA-7, Germany) filled with sand (0.7–1.2 mm grain size, Raiffeisen, Germany) and grown in a Vötsch chamber (22°C, 65% humidity, 16 h light). For single inoculations, seedlings of each genotype were individually inoculated with one of the selected 18 bacterial isolates or distilled water as control (Table S2 in File S1), while for the mixed inoculations, inoculation was conducted with a mixture of the 18 bacterial isolates (50 µL of each isolate at OD_600_  = 1). Two weeks after inoculation the length of the primary roots and the plant biomass were determined and the leaf surface area was analyzed using Adobe Photoshop C5 [74] and bacterial re-isolation was carried out as described above. Bacteria were identified by morphology and 16S rRNA sequencing.

### Phytohormone levels after elicitation of specialist-inoculated glasshouse-grown plants

Phytohormone production (JA, SA, and ET) was determined in EV plants inoculated with two bacterial isolates retrieved only from EV (*Pseudomonas frederiksbergensis* A176, *Pseudomonas koreensis* A21) and ir*AOC* plants (*Kocuria palustris* B56, *Kocuria marina* D102), respectively. After surface-sterilization, EV seeds were incubated in bacterial solution and germinated as described above. Plants were grown according to [44] and at day 30, fully-expanded young rosette leaves (+2 nodal position, source leaf [36]) were mechanically wounded with a pattern wheel and the puncture wounds immediately treated with 20 µL of 1∶5 diluted *M. sexta* oral secretion [75] or sterile distilled water. Leaves were harvested at 60 and 120 min after treatment. JA and SA levels were quantified with a UPLC-UV-ToF-MS according to [76]. For the determination of ET emissions, leaves were treated as above, and leaf discs (12 mm in diameter, 35.5±1 mg tissue) were punched from the mesophyll tissue on one side of the midvein, and placed in 4 mL transparent glass GC vials with screw lids and PTFE septa (Macherey-Nagel, Germany). Leaf discs were left to incubate for 5 h and ET was measured using a stop-flow set-up using a photoacoustic laser (ETD 300, Sensor Sense, and The Netherlands). Each vial was sampled for 5 min, which was sufficient to detect the entire peak of accumulated ET based on a stop-flow detached leaf method.

### Nucleotide sequence accession numbers

The sequencing data have been deposited in the European Nucleotide Archive- PRJEB4653, the isolates of the culture dependent approach are listed in Table S3.

## Results

### Primer pair 799F-1394R amplifies a minimum of chloroplast sequences with the highest diversity of bacterial sequences

**Figure 1:**
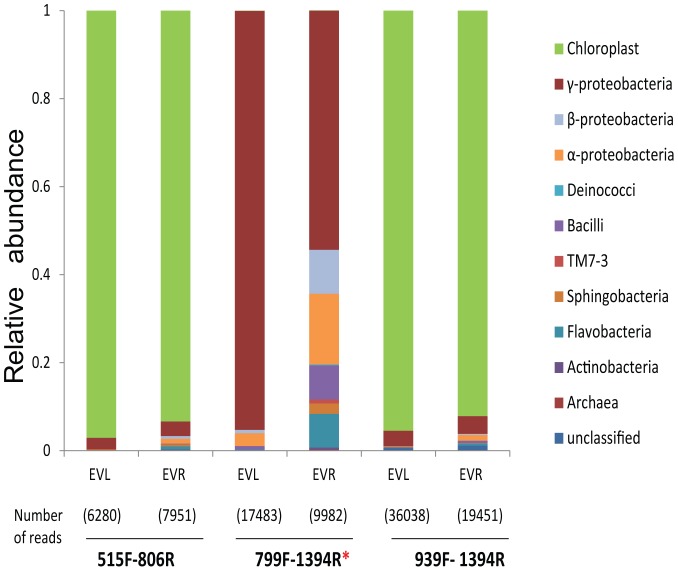
PCR primer pair 799F-1394R showed the lowest amplification of non-target chloroplast sequences comparing three different primer pairs. Relative abundance of bacterial classes recovered using three different primers (515F-806R, 799F-1394R & 939F- 1394R) from leaf and root samples of native field grown EV-genotype. Among these primers 799F-1394R amplified the lowest amount of reads matching with chloroplast sequences. Abbreviations: R, roots; L, leaves; *, PCR primer selected for further analysis.

Several primer combinations have been used to characterize the bacterial community by pyrosequencing. In order to find out which primer set had the highest specificity for bacterial sequences and retrieved the greatest diversity of taxa from our samples. We tested three different primer sets spanning the variable region of 16S rRNA from V4 to V9 (Figure S1A, [6]). For two primer sets, 515F-806R and 939F-1394R, between 94 and 97% of the reads were chloroplast sequences, and only 6 and 7 bacterial classes were amplified, respectively. With primer pair 799F-1394R less than 1% was chloroplast sequences (Figure 1) and reads associated with 9 bacterial classes were retrieved (Figure S1B). Shannon diversity index and Margalef species richness were higher for the sequences retrieved by the 799F-1394R primer pair compared to those retrieved by the two others (Figure S1C,D). Based on these results, we selected 799F-1394R primers for further analyses.

### Bacterial communities are independent of plant developmental stages

**Figure 2:**
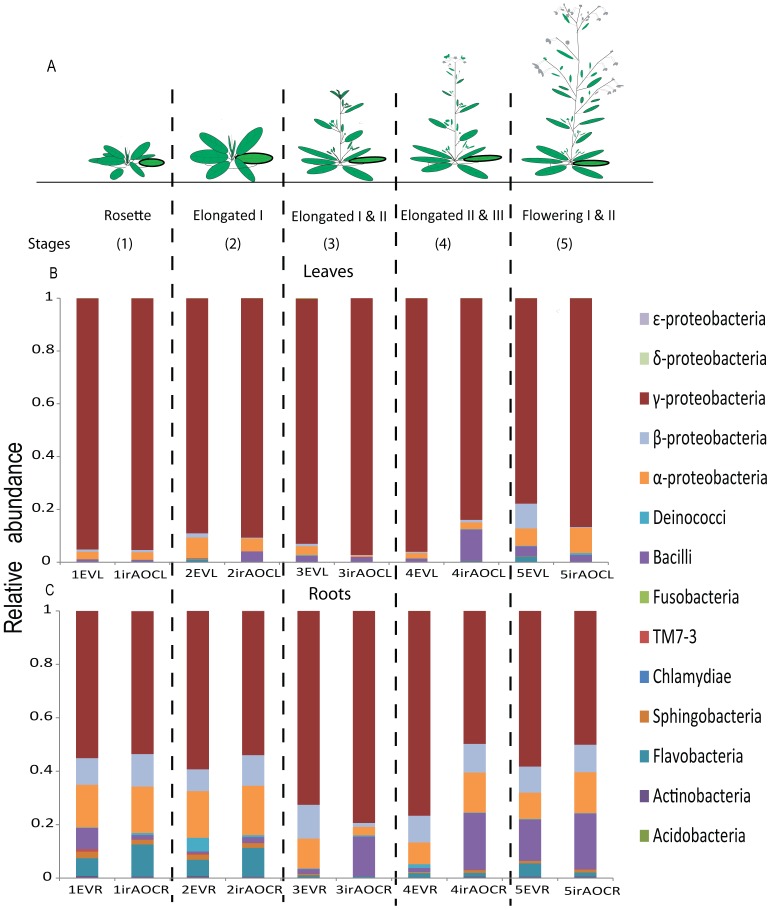
Native field grown EV and ir*AOC* leaf and root-associated bacterial communities (OTUs) are not influenced by developmental stages and jasmonic acid. In the culture-independent approach, at the class level relative abundance of operational taxonomic units (OTUs) of field-grown *N. attenuata* plants is independent of the developmental stages and the ability of the plant to produce jasmonates (JA). JA-producing empty vector (EV) plants and plants impaired in JA biosynthesis by silencing allene oxide cyclase (ir*AOC*) were grown in pairs at a field site in the plant's native habitat. Schematic representation of the plants' developmental stages at harvest **(A)**. Abundance of bacterial composition at the class level in EV and ir*AOC* leaves **(B)** and roots **(C)**. For abbreviations see Figure 1. All samples were rarefied to 6374 sequences.

To investigate the influence of developmental stages on the plants' root- and shoot-associated bacterial communities, we analyzed the bacterial communities of EV and ir*AOC* roots and leaves grown in their native habitat in Utah, USA by pyrosequencing. The dataset comprised 6,500–19,000 reads for each sample (Figure S2). To compare and minimize heterogeneity among samples, all samples were rarefied to 6,374 reads per sample. The RDP Bayesian classifier assigned all the sequences to 14 different bacterial classes (Figure 2B,C). γ, β, α- proteobacteria along with Bacilli and Flavobacteria were identified in all samples independently of genotype, tissue and harvest time. Furthermore, bacterial classes such as δ, ε, proteobacteria along with Fusobacteria, Chlamydiae, candidate division TM7-3 and Acidobacteria appeared sporadically throughout the different developmental stages of roots of both genotypes.

**Figure 3:**
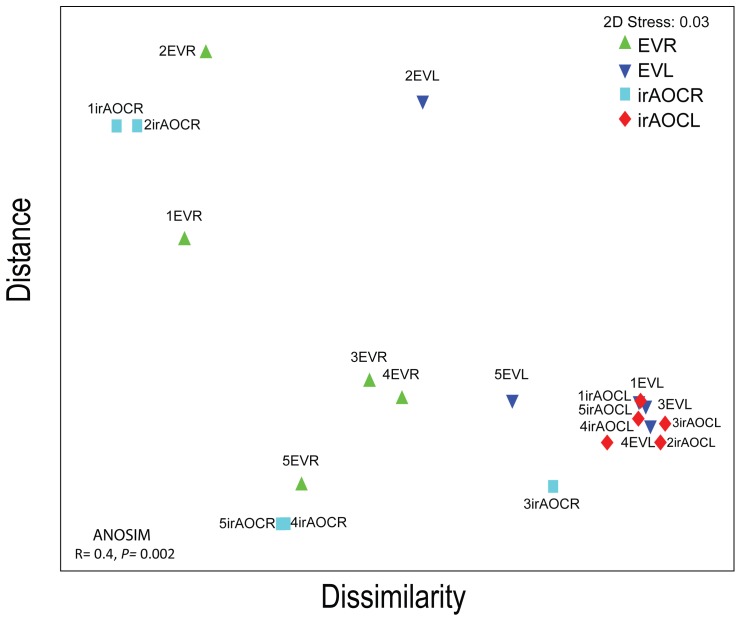
Based on the pyrosequencing results beta diversity of OTUs is influenced by tissues (leaves & roots) but not by genotypes (EV & ir*AOC*). In non-parametric multidimensional scaling (MDS) ordination, proximity of points reflects similarity. OTUs diversity among EV and ir*AOC* leaves and roots is highly similar, indicating bacterial communities are independent of JA and developmental stages but not tissues. Global ANOSIM among roots and leaves of both genotypes is significantly different (*p* = 0.002). MDS ordination and ANOSIM were determined by the weighted Uni-Frac distance metric based on OTUs rarefied to 6374 reads for each sample. Abbreviations: R, roots; L, leaves. Refer to Figure 2 for abbreviations and the experimental set-up in the field and harvest of plants.

The calculation of the weighted Uni-Frac beta-significance values, which identify pairs of samples that are significantly different from one other [70], indicated that root and leaf bacterial communities did not change significantly over development except for a few single time-points, which were only marginally different from one another (Table S4 in File S1). In order to visualize the similarity of bacterial communities among genotypes and tissues at the different developmental stages, a non-parametric multidimensional (MDS) ordination was constructed. The MDS plot shows that leaves are clustered closely together irrespective of developmental stages and genotypes, while roots showed an overall higher heterogeneity among samples, and samples of earlier developmental stages tend to group more closely together than from flowering plants independent of genotypes (Figure 3). Therefore we further evaluated if plant development like transition from rosette to flowering stages (young versus old) influences bacterial communities. We pooled the samples with stems not yet developed as young (rosette and elongated stage I) and elongated and flowering plants as old (I & II, elongated II & III and flowering I & II), because earlier studies showed that the JA outburst against insects dramatically shift from rosette to flowering transition [36]. However, neither alpha (Figure S3) nor beta diversity (ANOSIM, Table S5 in File S1) differed among these two developmental stages. We conclude that bacterial communities are largely independent of plant developmental stages irrespective of genotypes.

### JA biosynthesis does not change the overall bacterial communities

**Figure 4:**
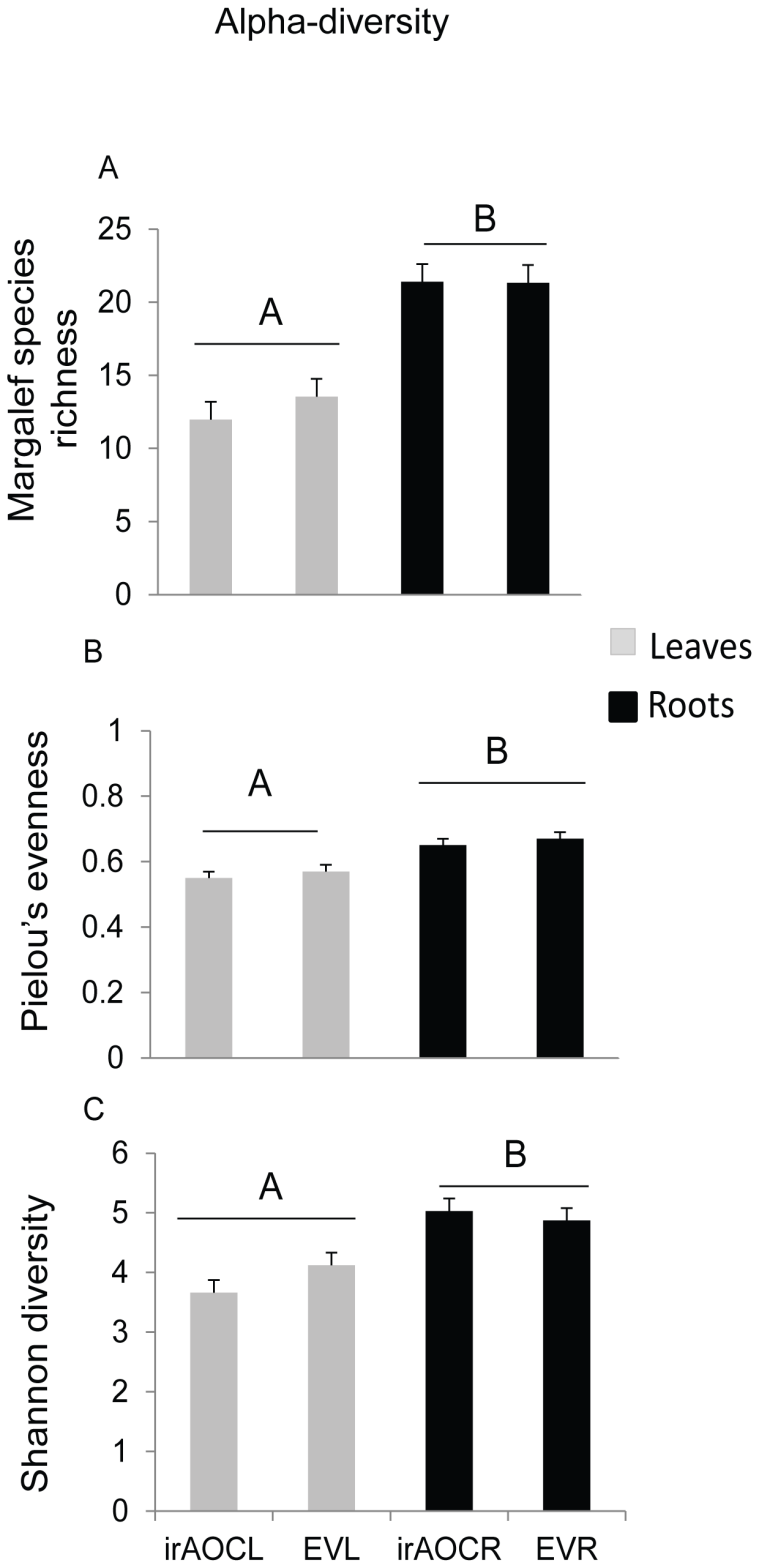
Alpha diversity of OTUs among leaves and roots of EV and ir*AOC* genotypes is significantly different. Based on the pyrosequencing results, alpha diversity indices such as Margalef's species richness (A), Pielou's evenness (B) and Shannon diversity (**C**) were significantly different among the ‘genotypes & tissues’ (EVL, ir*AOC*L, EVR, ir*AOC*R). Mean, ±SE, n = 5 subsampling (6374 sequences for each sample), different letters indicate significant differences, one-way ANOVA with Fisher's PLSD test; *P*<0.05.

In order to robustly evaluate whether endogenous JA levels influence bacterial communities we calculated the alpha and beta diversity indices of the leaf- and root-associated bacterial communities of EV and ir*AOC* genotypes. The Margalef's species richness, Pielou's evenness and Shannon diversity index were significantly different among ‘genotypes & tissues’ (Figure 4, Margalef's species richness ANOVA; F_3,16_ = 17.26, *p*<0.001, Pielou's evenness ANOVA; F_3,16_ = 5.67, *p* = 0.007, Shannon diversity ANOVA; F_3,16_ = 9.27, *p* = 0.0009); differences were due to discrepancies in bacterial communities among leaves and roots, but not genotypes. These findings are in accordance with the beta-diversity analysis of similarities (ANOSIM, Table 1) based on the weighted Uni-Frac distance matrix. The same results described here at 97% sequence identity were obtained when we analysed the data at the class and genera level (data not shown). We conclude that overall bacterial communities are independent of JA biosynthesis.

**Table 1:** Pairwise ANOSIM analysis among EV and ir*AOC* genotype tissues of the culture-independent approach.

Pairwise Tests	Culture-independent	
Groups	R-Statistic	*P*
EVR and EVL	0.51	0.02
EVR and ir*AOC*L	0.83	0.008
ir*AOC*R and EVL	0.45	0.02
ir*AOC*R and ir*AOC*L	0.71	0.008
EVL and ir*AOC*L	0.06	0.73
EVR and ir*AOC*R	0.04	0.66
Global	0.45	0.002

Abbreviations: R, roots; L, leaves and for the experimental set up and approach see Figure 2.

### Roots and leaves harbor distinct bacterial communities

To further elucidate the differences in bacterial communities among root and leaf samples we examined the abundance of 8 core OTUs at the class level which were present in roots and leaves, and covered ≥7–<90% of the total abundance across all samples. Abundance of five classes (Actinobacteria, Sphingobacteria, Flavobacteria, β, α- proteobacteria) were higher in roots compared to leaves, while γ-proteobacteria dominated the bacterial community of leaves. In contrast, candidate division TM7-3 and Acidobacteria bacterial classes were only retrieved from roots across all developmental stages irrespective of genotypes. At genera level, the abundance of 14 OTUs was significantly different among roots and leaves (Table S6 in File S1). In summary, both alpha and beta diversity (Figure 3&4 and Table 1) variation analysis strongly suggest that root- and leaf associated bacterial communities are determined by the tissue type but not by the JA signaling capacity of the plants.

### At the genera level 21 OTUs differ among plant genotypes

**Figure 5:**
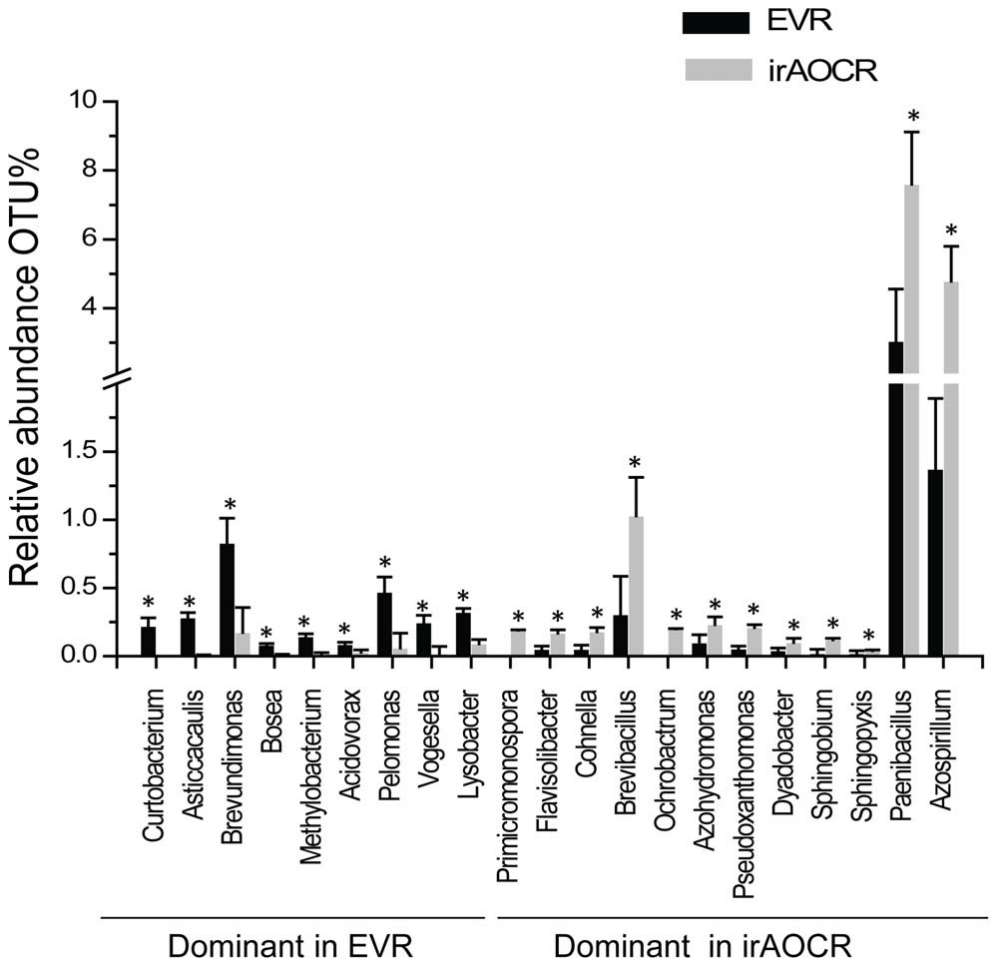
At the genera level, 21 OTUs differ significantly among the roots of the two genotypes (EVR and ir*AOC*R). OTU significance test was carried out with rarefied 6374 reads and OTUs which were significantly different were binned at the genera level. Mean, ±SE, n = 5, one-way ANOVA with Fisher's PLSD test; *P<0.05.

A more detailed analysis indicated that at the genera level, 21 OTUs differed significantly between the two genotypes; 9 OTUs were retrieved in higher abundance from EV roots and 12 from ir*AOC* roots (n = 5, *p*<0.05, Fisher's PLSD, Figure 5). In particular, OTUs corresponding to *Paenibacillus* and *Azospirillum* were much more abundant in ir*AOC* than EV roots. The differences among the genotypes were only found in roots, not leaves. Based on these results we conclude that roots harbor a greater diversity of bacterial communities compared to leaves, and the diversity of bacterial communities is largely independent of JA signaling, though the plants' capacity to produce JA may influence the occurrence and abundance of particular genera.

### Putative EV and ir*AOC* specialist and generalist isolates did not show colonization specificity under *in-vitro* conditions

In addition to pyrosequencing, we employed a culture-dependent approach using the same plant material, because only cultured bacteria allow a functional analysis and further in-depth study of putative differences in colonization patterns. A total of 414 bacterial isolates were retrieved from surface-sterilized roots and leaves of both genotypes. Based on the 16S rRNA gene sequences, the isolates were assigned to 131 different species and 6 classes (116 and 89 isolates from EV, 107 and 102 from ir*AOC* roots and leaves, respectively, Table S3). A comparison of the genotypes revealed that 42 species (66 isolates) were only isolated from ir*AOC* plants, and we tentatively considered these as putative ir*AOC* specialists. Similarly, 51 species (121 isolates) were only recovered from EV plants (putative EV specialists), while 38 species (227 isolates) were found in both genotypes, and these we considered as generalists. Furthermore, members of the genera *Pseudomonas* colonized more frequently in EV plants (58 isolates) than ir*AOC* plants (22 isolates) over all developmental stages; whereas, the genera *Kocuria* was only retrieved from ir*AOC* plants (11 isolates, Table S3). Interestingly, eight genera isolated by the culture-dependent approach were also recovered in higher abundance from EV or ir*AOC* roots in the pyrosequencing approach (Figure 5).

To evaluate the reproducibility of the observed genotype-specific colonization patterns found in the field, we performed *in-vitro* re-colonization assays by inoculating seedlings either with single bacterial isolates or with a mixture of all bacterial isolates used in the single inoculations. We used the mixed inoculation procedure to recapitulate a more natural situation, and to evaluate if plants only recruit specific bacterial isolates from a mixture of cultures. Isolates were selected based on a) their difference in abundance in the two genotypes in the pyrosequencing and the culture-dependent approach and b) the beneficial effects of some species described in literature (Table S2 in File S1).

**Figure 6:**
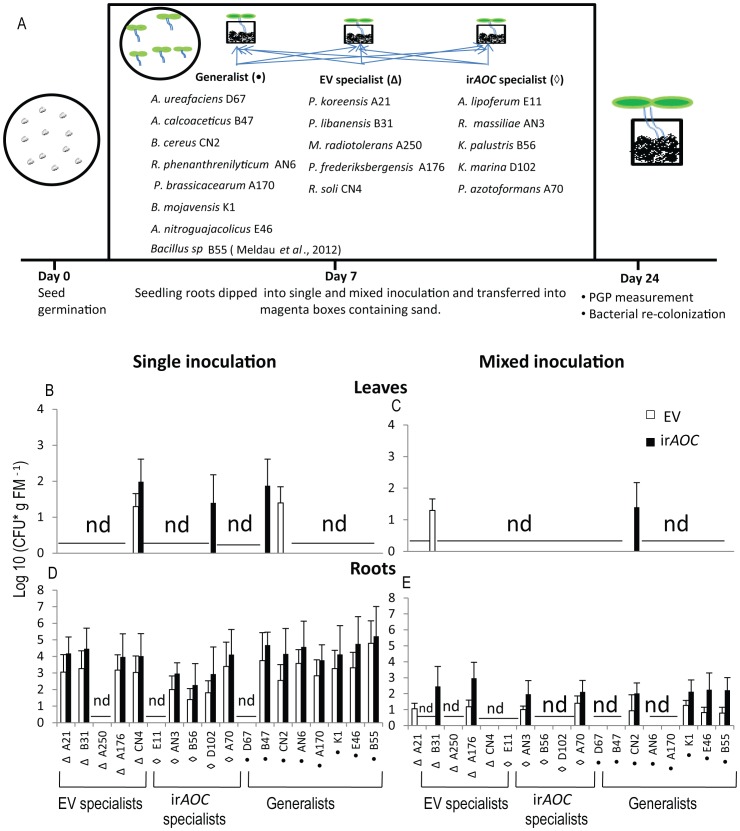
Cultured putative EV and ir*AOC* genotype specialist bacterial isolates did not colonize specifically to either genotype under *in-vitro* conditions. Bacterial colonization is independent of genotypes. Leaf (B,C) and root (D,E) colonization of EV and ir*AOC* plants grown under *in-vitro* conditions for 24 days after single or mixed inoculation of EV and ir*AOC* seedlings. Experimental set-up and list of species used (A): bacteria selected were either isolated only from EV or ir*AOC* genotypes (putative specialists) or generalists, isolated from both genotypes. Seven day old seedlings were inoculated by dipping their roots for 1 min into 1 mL of bacterial suspension of a constant OD = 1 at 600 nm of a single isolate or a mixture of all isolates (50 µL each of isolate). The identity of bacterial isolates was confirmed by morphology and 16S rRNA sequencing. Mean (±SE), CFU, colony-forming units; nd, not detected; FM, fresh mass; n = 6.

Overall, single and mixed inoculations with most bacterial isolates resulted in a poor colonization of leaves compared to roots of both genotypes (Figure 6B,C). Only 4 species were able to colonize both ir*AOC* and EV leaves. The colonization pattern of leaves and roots was independent of the putative specialist and generalist behavior observed in the field. In summary, under *in-vitro* conditions we could not confirm the apparent putative genotype-specific colonization behavior observed by some bacterial isolates in the field. The results are consistent with the hypothesis that bacterial colonization of plants is not primarily shaped by JA signaling, and depends upon the availability of individual bacterial isolates to infect plants in the soil at the plant's particular planting site.

### Elicitation of JA, SA and ET did not differ among EV and ir*AOC* plants inoculated with the specialist taxa

We not only evaluated the effects of the plant's ability to elicit endogenous JA-signaling on its bacterial community but also if bacterial colonization alters the phytohormone levels in elicited plants. We inoculated EV seeds with two *Pseudomonas* species (*P. koreensis* A21, *P. frederiksbergensis* A176) isolated only from EV plants in the field and two *Kocuria* species found only in ir*AOC* plants (*K. palustris* B56, *K.marina* D102, Figure S4). These species were selected among other specialists because they were more abundant at different developmental stages, and were retrieved from both roots and leaves. We measured changes in SA, JA and ET production in leaves of rosette stage EV plants after elicitation by mechanical wounding followed by application of oral secretion of *M. sexta* (OS_MS_). The production of all three phytohormones significantly increased after elicitation with OS_MS_ compared to wound and water elicitation only (ANOVA; JA, F_9,20_ = 26.15, *p*<0.001. SA, F_9,20_ = 6.02, *p*<0.001. ET, F_9,20_ = 3.54, *p*<0.01) (Figure S5). However, there was no significant difference in phytohormone levels between the inoculation with the four bacterial isolates and water-treated controls (ANOVA; JA, F_4,12_ = 0.328, *p* = 0.85. SA, F_4,12_ = 2.143, *p* = 0.15. ET, F_4,12_ = 0.31, *p* = 0.86, Figure S5). Thus, inoculation of EV plants with field-observed putative specialist bacterial isolates did not influence the OS-elicitation of JA, SA and ET.

## Discussion

In nature, plants are subjected to various biotic and abiotic stresses throughout their development which in turn may influence the composition of the bacterial communities [77], [78]. In this study, we conducted a comprehensive pyrosequencing and culturing analysis of the temporal changes in bacterial communities of leaves and roots of plants grown in their native habitat. Additionally, we investigated the effects of JA signaling, and its associated defenses on the development of the plant's bacterial community because the literature on this topic is sparse and contradictory [34], [35]. Our approach differs from previous studies which were either conducted under controlled glasshouse conditions [6], [39] or used only culture-dependent techniques [34], [35], [79]. Our results not only support the results of earlier studies [6], [35], [39], but also provide new insights into root and shoot bacterial communities exposed to their natural environment.

Culture-independent approaches based on pyrosequencing are influenced by the primers and sequencing depth [58]. We tested three different primers, and sequences retrieved from two primer sets were largely chloroplast DNA, while 799F primer minimized the contamination of chloroplast DNA and excluded Cyanobacteria [12], [59]. According to Ghyselinck *et al*. [18] this primer matches only with 78.5% of bacteria based on SILVA SSU ref 113 NR database, however, in our primer test 799F sequence reads resulted in higher alpha diversity and were assigned to more phyla (Figure S1) than the reads from the two other commonly used primers.

We tested the hypothesis that ontogeny alters community composition of roots and leaves, because inducible defense signaling mediated by JA is known to change dramatically at the rosette-flowering transition in *N. attenuata* [36]. Earlier studies indicated significant differences in bacterial populations over season in roots and leaves of soybean and rice [15], [21], [79], [80]. For soybean plants it was shown that the density of bacteria decreased with age from vegetative growth to senescence [79], while diversity of *Pseudomonas* species decreased over development starting from young to senescent stages [79]. However, those studies only analyzed the culturable communities, while in the present study, based on deep sequencing of the 16S rRNA region, we found that bacterial communities are independent of plant developmental stages, and the weighted Uni-Frac beta significance test only showed a few pairs with marginally significant differences (Table S4 in File S1). Similarly, a comparison of alpha (Figure S3) and beta (Table S5 in File S1) diversity indices of the rosette-flowering transition indicated that bacterial communities are independent of developmental stages. Our findings are in agreement with the extensive study of Lundberg *et al*. [6] who investigated the influence of plant developmental stages of young versus old tissues on bacterial communities of *Arabidopsis*, which were grown in two different soil types in the glasshouse. This study also demonstrates that bacterial communities do not alter over developmental stages. We conclude that plant development does not have a major effect on community composition in native field grown *N. attenuata* plants.

In contrast to development, tissue type has a major effect on the bacterial community composition (Figure 3&4, Table 1), which was also observed for field grown soybean [79] and *Arabidopsis* [12] plants. In accordance with previous studies, the dominating bacterial class in leaves was γ- proteobacteria (Figure 2) [12]. At the genera level two OTUs, namely *Serratia* and *Enterobacter* heavily dominated the phyllosphere, while in many other studies *Pseudomonas* was the dominating genera [81], [82]. Overall, roots recruited more diverse bacterial communities than the leaves (Figures 3, 4). Genera belonging to α- proteobacteria such as *Rhizobium* and *Azospirillum* (15–25% abundance) which are well-known to dominate the root communities of nitrogen fixing plants may have a positive effect on plant growth and health [21], [83]. Based on these findings we assume that a core bacterial community is recruited from the soil, but roots and leaves provide different niches for bacterial growth. The roots' higher diversity may be due the secretion of root exudates and the direct contact of the roots with the soil microbiome [11], [15], while bacterial communities of the leaves are additionally influenced by rain splashing of the soil, dust or wind [79].

The phytohormone JA is known to play a central role in plant defense against leaf-chewing herbivores, but it is also involved in induced systematic resistance (ISR) against pathogens [33]. However, influences of JA on bacterial communities at different plant developmental stages have so far received little attention [34], [84]. Unlike, this study, Doornbos *et al*. [35] showed that the JA-response mutant *jar1* harbored significantly lower numbers of culturable bacteria compared to Co1-0 wild type, while a different study with the same *Arabidopsis* ecotype and a transgenic line impaired in the production of JA biosynthesis could not find any difference in the culturable leaf-associated bacterial communities [34]. A recent field study with 27 different maize genotypes also revealed small, but significant differences in diversity indices among genotypes [58]. Native *N. attenuata* plants are genetically diverse and individual plants of a population accumulate different amounts of JA after herbivore attack [47]. It would not be surprising if natural variation in JA accumulation also leads to the colonization with different bacterial communities that help a plant to compensate for JA-deficiencies, as we had previously demonstrated for ET-deficient plants [74], [85]. However, our study did not support this expectation. Despite the differences in primary and secondary metabolites such as sugars, starch and nicotine between EV and ir*AOC* genotypes [86], results of our field study did not reveal any significant differences in alpha diversity indices (Figure 4), beta diversity variance or MDS plots (Figure 3, Table 1), though at the genera level some OTUs were significantly different between roots of EV and ir*AOC* plants (Figure 5). Unfortunately, up to now it is not possible to validate the pyrosequencing results experimentally. Therefore, we used selected isolates from the culture-dependent approach to test if they specifically colonize one of the genotypes. None of the isolates tested showed a preferred colonization of EV or ir*AOC* (Figure 6). In addition, EV and ir*AOC* specialist treatment of plants did not alter the OS-elicited accumulation of phytohormones (JA, SA, ET; Figure S5). These findings strongly suggest that neither the plants' capacities to produce JA, nor JA-elicited primary and secondary metabolites, play a major role in shaping root- and leaf-associated bacterial communities. However, it cannot be ruled out that JA has an effect on some genera which could not be retrieved by the culture-dependent approach. Furthermore, the *in-vitro* assay may not allow selective root colonization and recruitment of selected species.

This study demonstrates that the recruitment of root- and leaf-associated bacterial communities by *N. attenuata* in its native habitat is independent of the developmental stages and JA signaling, but is mostly driven by the composition of the community that the plant first comes in contact with when it germinates from the seed bank and as plants grow, different tissues (roots and leaves) established distinct bacterial communities. The colonization of plants by bacterial communities appears to be opportunistic, and mainly depending on the local soil microbe population. However, under specific circumstances (e.g. biotic and abiotic stresses) these opportunistic interactions may become mutualistic and help plants to adapt to these stresses, as has been recently shown by Meldau *et al*. [74], [85]. The mechanisms responsible for these opportunistic mutualisms need further investigation.

## Supporting Information

Figure S1 **Base position of primers on 16S rRNA and primer comparison with regard to diversity of bacterial classes covered.** The variable base positions on the 16S rRNA gene of the three different primer pairs tested (A, 515F-806R, 799F-1394R & 939F- 1394R). The number of bacterial classes (B), Margalef species richness (C) and Shannon diversity (D) recovered by primer 799F- 1394R was higher than for the two other primer pairs tested. Results are based on a pooled EV leaf and root samples. Abbreviations: R, roots; L, leaves; *, primer pairs selected for further analysis.
(TIF)

Figure S2 **Rarefaction curves based on pyrosequencing reads, describing the observed number of operational taxonomic units (OTUs) as a function of the sequencing reads per each root and leaf samples. The OTU richness is higher in roots than leaves.** Partial 16S rRNA gene sequences were pooled into single OTUs at the cut off value of 97% similarity. For abbreviations see Figure S1, the vertical line indicates the number of reads subsampled from each sample (6374 reads) for normalization.
(TIF)

Figure S3 **Root and leaf bacterial communities are independent of developmental stages.** Alpha diversity indices were not significantly different among young and old developmental stages of EV and ir*AOC* genotypes. Samples without stem (Rosette and elongated stage I) were merged as young and plants with elongated stems and flowering (elongated I & II, elongated II & III and flowering I & II) pooled as old. Mean, ±SE, n = 2–3 different letters indicate significant differences, one-way ANOVA with Fisher's PLSD test; *P*<0.05.
(TIF)

Figure S4 **EV and ir** ***AOC*** **genotype putative bacterial specialist isolates identified by the culture-dependent technique.** Two *Pseudomonas* species (*P. frederiksbergensis*A176 (**Δ**), *P. koreensis*A21 (**Δ**)) were only isolated from EV plants, and two *Kocuria* species (*K. palustris*B56 (**◊**) and *K. marina* D102 (**◊**)) only from ir*AOC* field-grown plants in high numbers. These taxa were considered as putative specialists on their respective hosts. Mean (±SE), nd, not detected; n = 19. For the experimental set-up, harvest of plants and isolation of bacteria see Figure 2.
(TIF)

Figure S5 **Inoculation with putative bacterial specialist isolates did not influence the different phytohormone elicitation.** Phytohormone elicitation is independent of putative bacterial specialist inoculation. Elicitation of jasmonic acid (JA, B), salicylic acid (SA, C) and ethylene (ET, D) was not significantly different among plants inoculated with the specialist bacterial isolates from EV and ir*AOC* plants. Experimental design (A): Plants were seed-inoculated with different bacterial strains by incubating the seeds overnight in bacterial suspension (OD600 = 1). Rosette-stage EV leaves were wounded with a fabric pattern wheel followed by the application of oral secretion (OS) of *Manduca sexta* (wound + OS_MS_, 20 µL) or water (Wound + water) to punctured wounds to faithfully mimic *M. sexta* larva attack. JA levels were measured 60 min and salicylic acid 120 min after treatment. Ethylene accumulated for 5 h after elicitation. Mean ±SE; FM, Fresh mass; n = 5.
(TIF)

File S1 **Table S1,S2, S4, S5, S6: Table S1. Average rosette diameter and stalk length of native field grown** ***N. attenuata*** **at the time of harvest. Table S2. Cultured putative specialist and generalist bacterial isolates used in this study. Table S4. Uni-Frac beta diversity is not significantly different among developmental stages of EV and ir** ***AOC*** **genotypes tissues indicating that bacterial communities are independent of developmental stages. Table S5. Pairwise ANOSIM did not differ significantly among rosette (young) and elongated, flowering (old) developmental stages. Table S6. List of OTUs significantly different among EV and ir** ***AOC*** **leaves and roots at genera (or higher) level retrieved by pyrosequencing.**
(PDF)

Table S3 **Abundance of culturable bacterial species from surface-sterilized roots and leaves of EV and ir** ***AOC*** **genotypes over different plant developmental stages.**
(XLSX)
