# Supplementary figures and images for: Analysis of Plant-Bacteria Interactions in Their Native Habitat: Bacterial Communities Associated with Wild Tobacco Are Independent of Endogenous Jasmonic Acid Levels and Developmental Stages
Source: PLoS One. 2014 Apr 11;9(4):e94710. doi: 10.1371/journal.pone.0094710 (PMC3984252; doi:10.1371/journal.pone.0094710)

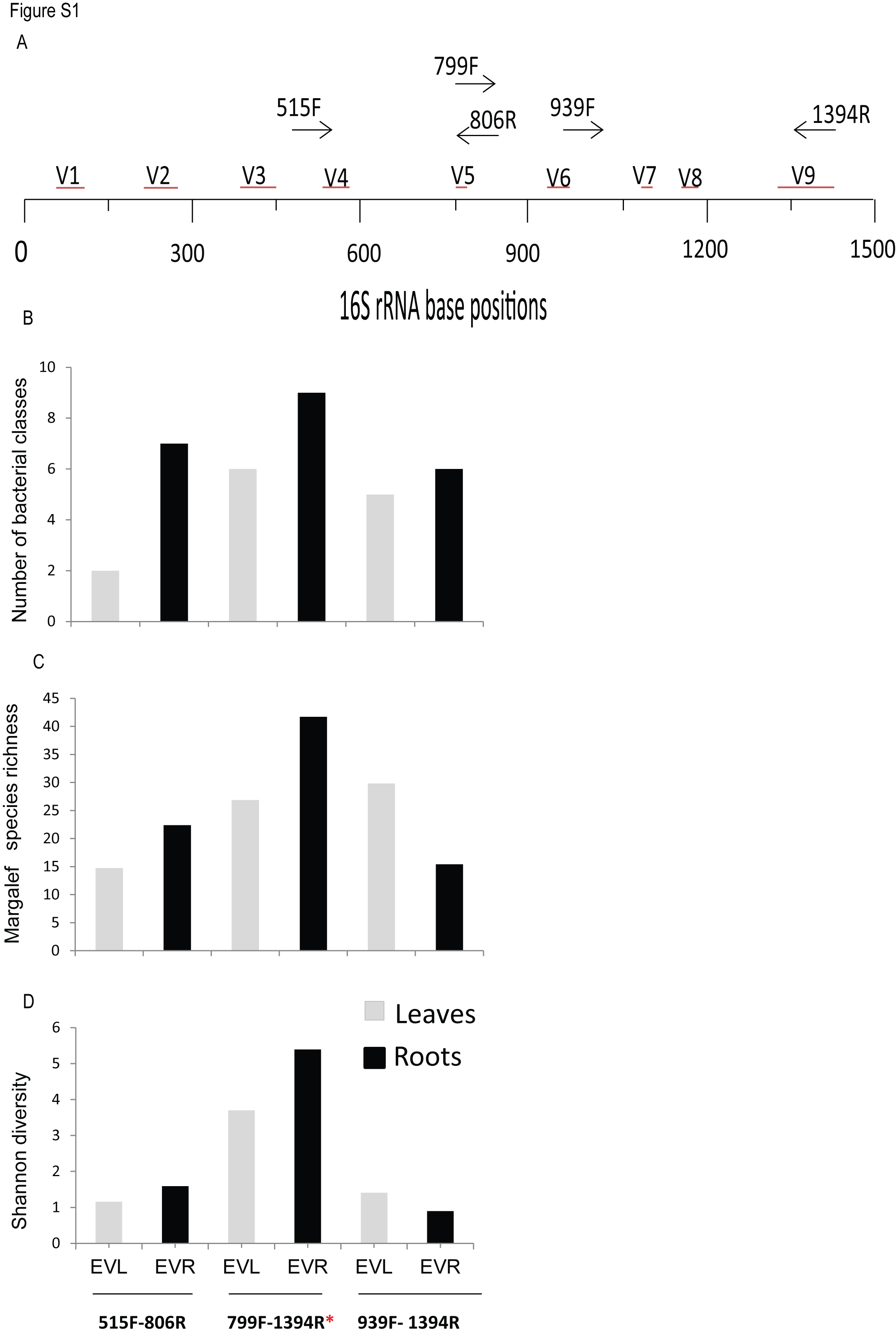

Supplement: Figure S1 — Base position of primers on 16S rRNA and primer comparison with regard to diversity of bacterial classes covered. The variable base positions on the 16S rRNA gene of the three different primer pairs tested (A, 515F-806R, 799F-1394R & 939F- 1394R). The number of bacterial classes (B), Margalef species richness (C) and Shannon diversity (D) recovered by primer 799F- 1394R was higher than for the two other primer pairs tested. Results are based on a pooled EV leaf and root samples. Abbreviations: R, roots; L, leaves; *, primer pairs selected for further analysis. (TIF) [file pone.0094710.s001.tif]

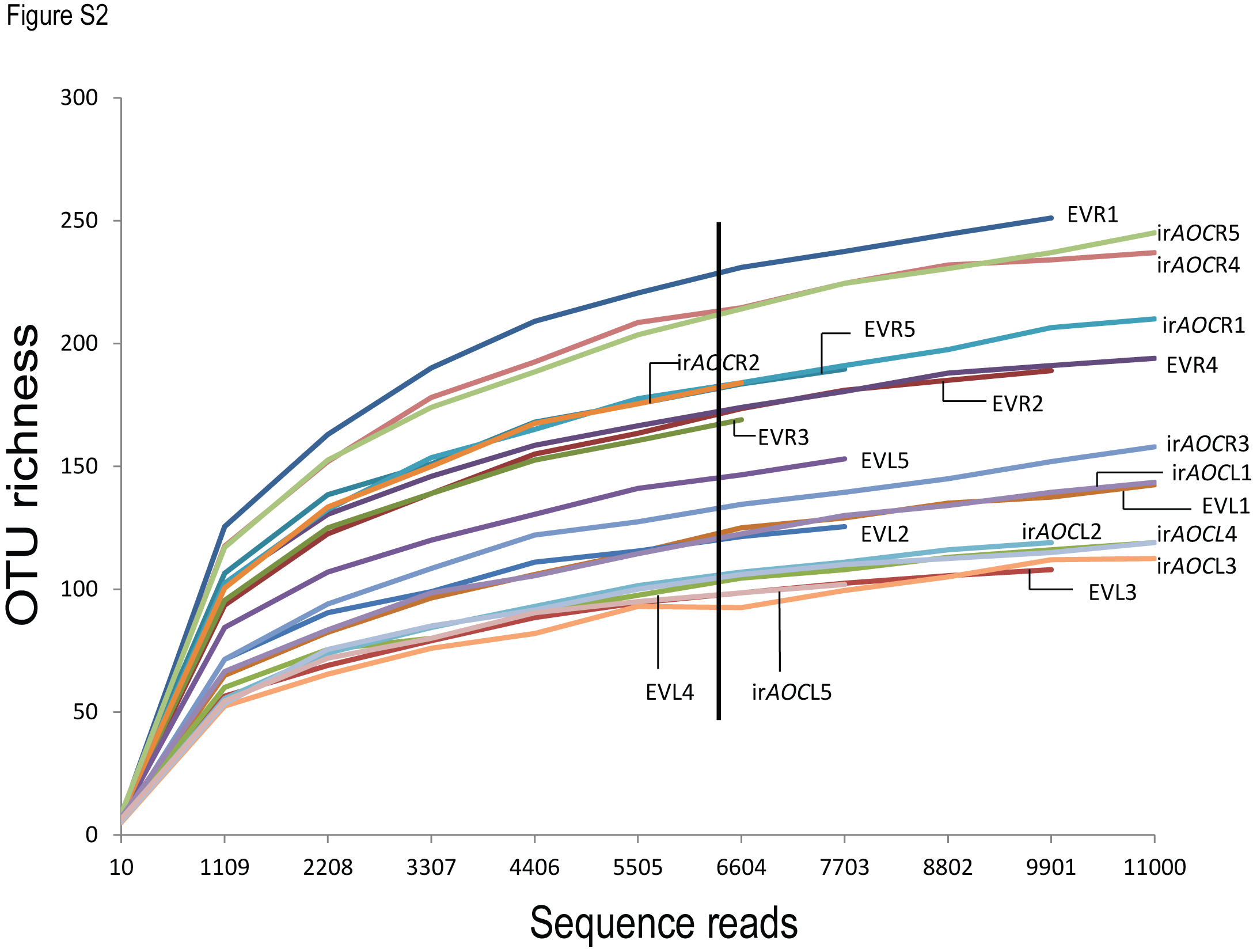

Supplement: Figure S2 — Rarefaction curves based on pyrosequencing reads, describing the observed number of operational taxonomic units (OTUs) as a function of the sequencing reads per each root and leaf samples. The OTU richness is higher in roots than leaves. Partial 16S rRNA gene sequences were pooled into single OTUs at the cut off value of 97% similarity. For abbreviations see Figure S1, the vertical line indicates the number of reads subsampled from each sample (6374 reads) for normalization. (TIF) [file pone.0094710.s002.tif]

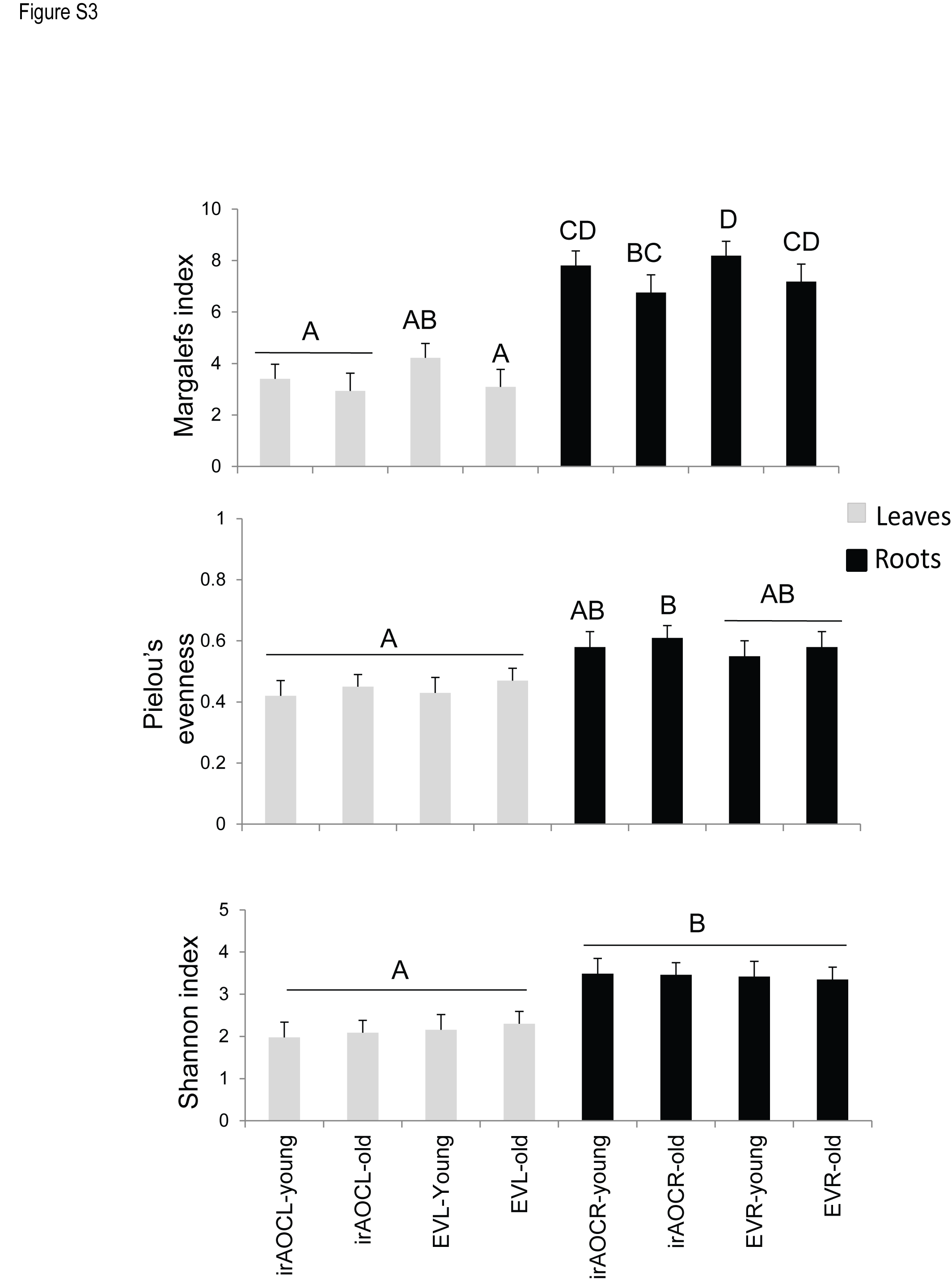

Supplement: Figure S3 — Root and leaf bacterial communities are independent of developmental stages. Alpha diversity indices were not significantly different among young and old developmental stages of EV and irAOC genotypes. Samples without stem (Rosette and elongated stage I) were merged as young and plants with elongated stems and flowering (elongated I & II, elongated II & III and flowering I & II) pooled as old. Mean, ±SE, n = 2–3 different letters indicate significant differences, one-way ANOVA with Fisher's PLSD test; P<0.05. (TIF) [file pone.0094710.s003.tif]

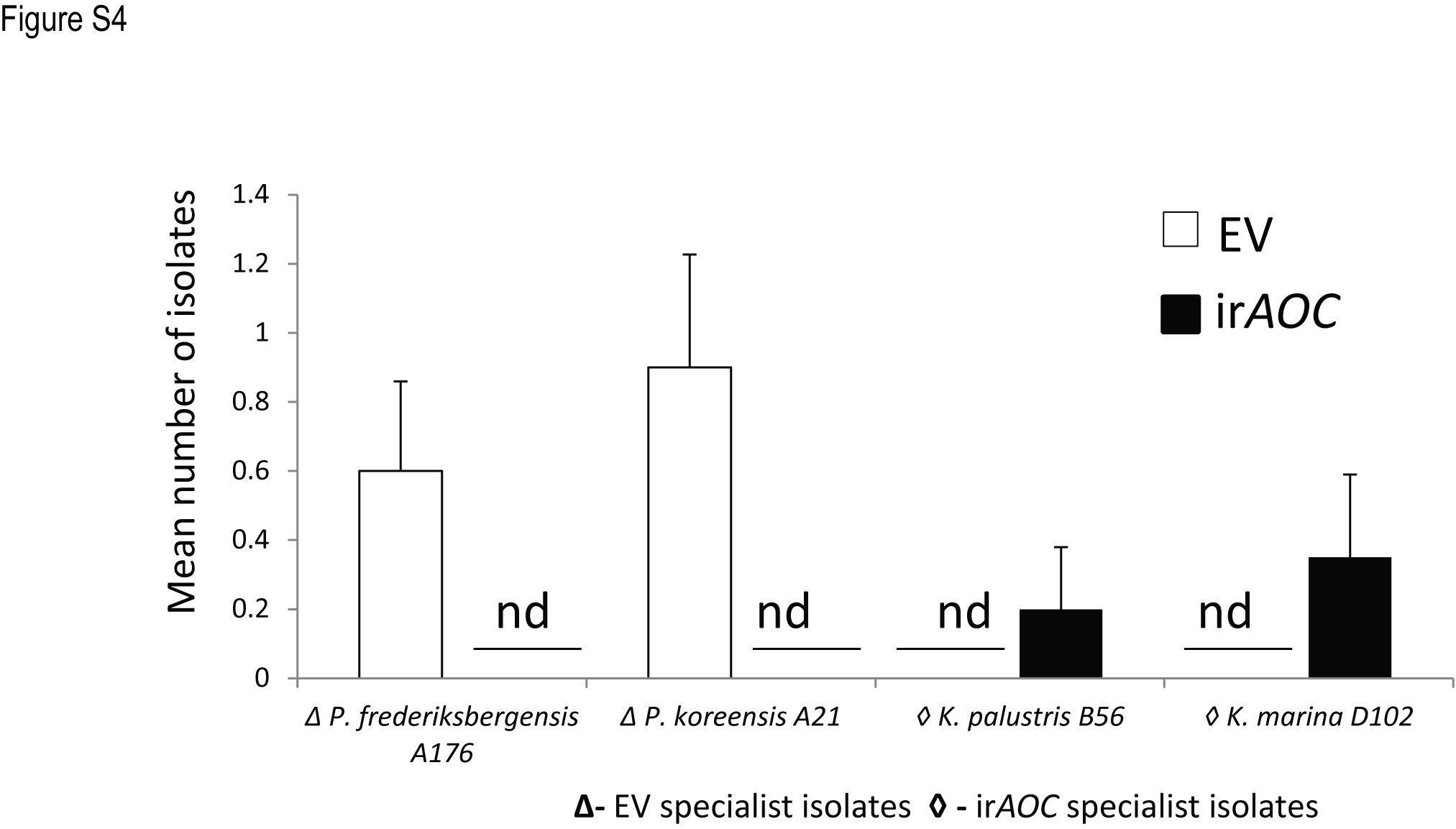

Supplement: Figure S4 — EV and ir AOC genotype putative bacterial specialist isolates identified by the culture-dependent technique. Two Pseudomonas species (P. frederiksbergensisA176 (Δ), P. koreensisA21 (Δ)) were only isolated from EV plants, and two Kocuria species (K. palustrisB56 (◊) and K. marina D102 (◊)) only from irAOC field-grown plants in high numbers. These taxa were considered as putative specialists on their respective hosts. Mean (±SE), nd, not detected; n = 19. For the experimental set-up, harvest of plants and isolation of bacteria see Figure 2. (TIF) [file pone.0094710.s004.tif]

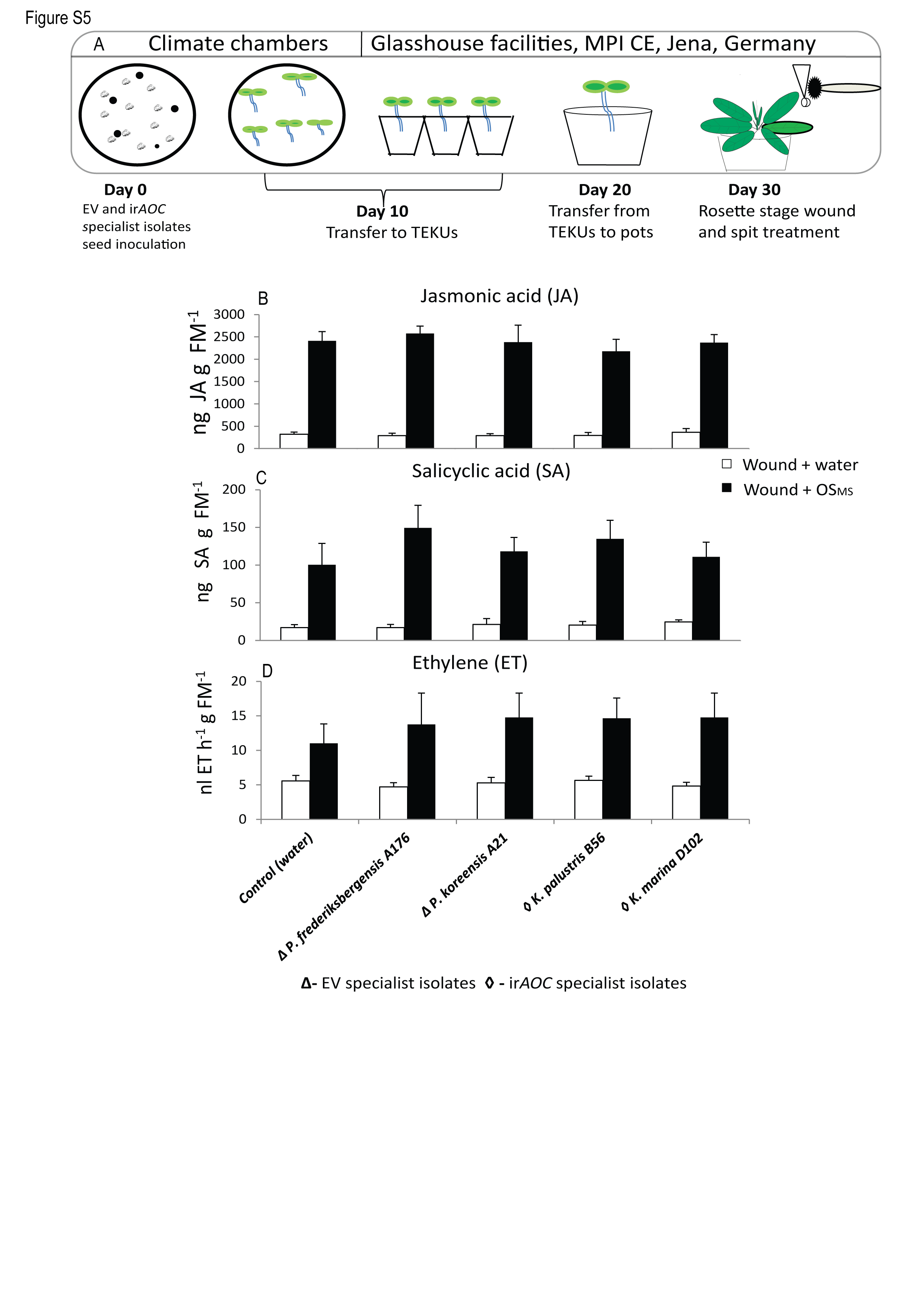

Supplement: Figure S5 — Inoculation with putative bacterial specialist isolates did not influence the different phytohormone elicitation. Phytohormone elicitation is independent of putative bacterial specialist inoculation. Elicitation of jasmonic acid (JA, B), salicylic acid (SA, C) and ethylene (ET, D) was not significantly different among plants inoculated with the specialist bacterial isolates from EV and irAOC plants. Experimental design (A): Plants were seed-inoculated with different bacterial strains by incubating the seeds overnight in bacterial suspension (OD600 = 1). Rosette-stage EV leaves were wounded with a fabric pattern wheel followed by the application of oral secretion (OS) of Manduca sexta (wound + OSMS, 20 µL) or water (Wound + water) to punctured wounds to faithfully mimic M. sexta larva attack. JA levels were measured 60 min and salicylic acid 120 min after treatment. Ethylene accumulated for 5 h after elicitation. Mean ±SE; FM, Fresh mass; n = 5. (TIF) [file pone.0094710.s005.tif]
