# Supplementary material for: Analysis of Plant-Bacteria Interactions in Their Native Habitat: Bacterial Communities Associated with Wild Tobacco Are Independent of Endogenous Jasmonic Acid Levels and Developmental Stages
Source: PLoS One. 2014 Apr 11;9(4):e94710. doi: 10.1371/journal.pone.0094710 (PMC3984252; doi:10.1371/journal.pone.0094710)
Supplement: File S1 — Table S1,S2, S4, S5, S6: Table S1. Average rosette diameter and stalk length of native field grown N. attenuata at the time of harvest. Table S2. Cultured putative specialist and generalist bacterial isolates used in this study. Table S4. Uni-Frac beta diversity is not significantly different among developmental stages of EV and ir AOC genotypes tissues indicating that bacterial communities are independent of developmental stages. Table S5. Pairwise ANOSIM did not differ significantly among rosette (young) and elongated, flowering (old) developmental stages. Table S6. List of OTUs significantly different among EV and ir AOC leaves and roots at genera (or higher) level retrieved by pyrosequencing. (PDF) [file pone.0094710.s006.pdf]

Table S1. Average rosette diameter and stalk length of native field grown *N. attenuata* at the time of harvest.

Stages	Rosette diameter (cm)		Stalk length (cm)	
	EV	ir*AOC*	EV	ir*AOC*
Rosette (1)	9.3	9.5	0	0
Elongated I (2)	13	17	5	8
Elongated I &II (3)	15	18	14	25
Elongated II & III (4)	28	31	21	36
Flowering I & II (5)	38	40	40	54

N=3-5.

Table S2. Cultured putative specialist and generalist bacterial isolates used in this study.

Isolates	Nearest type strains	Characteristics	CFU (OD600=1)	References
A21	*Pseudomonas koreensis* Ps 9-14^{T} (AF468452)	Specialist for EV	28 X 10^{-7}	This study
B31	*Pseudomonas libanensis* CCUG 43190^{T} (AF057645)	Specialist for EV	40 X 10^{-7}	This study
A250	*Methylobacterium radiotolerans* CBMB20^{T} (AY683045)	Specialist for EV	24X 10^{-5}	This study
A176	*Pseudomonas frederiksbergensis* JAJ28^{T} (AJ249382)	Specialist for EV	12 X10^{-7}	This study
CN4	*Rhizobium soli* KCTC 12873^{T} (EF363715)	Specialist for EV	12 X 10^{-6}	This study
E11	*Azospirillum lipoferum* ATCC 29707^{T} (Z29619)	Specialist ir*AOC*	40 x10^{-5}	This study
AN3	*Rhizobium massiliae* KCTC 12121^{T} (AY341343)	Specialist ir*AOC*	40 X 10^{-4}	This study
B56	*Kocuria palustris* DSM 11925^{T} (Y16263)	Specialist ir*AOC*	11 X 10^{-6}	This study
D102	*Kocuria marina* KMM 3905^{T} (AY211385)	Specialist ir*AOC*	13 X 10^{-6}	This study
A70	*Pseudomonas azotoformans* CCUG 12536^{T} (D84009)	Specialist ir*AOC*	43 X 10^{-7}	This study
D67	*Arthrobacter ureafaciens* ATCC 7562^{T} (X80744)	Generalist for both genotypes	12 X 10^{-7}	This study
B47	*Acinetobacter calcoaceticus* ATCC 23055^{T} (AJ888983)	Generalist for both genotypes	70 X 10^{-7}	This study
CN2	*Bacillus cereus* ATCC 14579^{T} (AE016877)	Generalist for both genotypes	17 X 10^{-7}	This study
AN6	*Rhizobium phenanthrenilyticum* DSM 21882^{T} (FJ743436)	Generalist for both genotypes	80 X 10^{-4}	This study
A170	*Pseudomonas brassicacearum* DSM 13227^{T} (AF100321)	Generalist for both genotypes	90 X 10^{-7}	This study
K1	*Bacillus mojavensis* ATCC 51516^{T} (AB021191)	Generalist for both genotypes	30 X 10^{-7}	This study
E46	*Arthrobacter nitroguajacolicus* DSM 15232^{T} (AJ512504)	Generalist for both genotypes	16 X 10^{-7}	This study
B55	*Bacillus megaterium* ATCC 14581^{T} (D16273)	Generalist for both genotypes	20 X 10^{-5}	Meldau et al., 2012.

OD, optical density at 600nm; CFU, colony forming units.

Table S4. Uni-Frac beta diversity is not significantly different among developmental stages of EV and ir*AOC* genotypes tissues indicating that bacterial communities are independent of developmental stages.

Samples	1EVL	2EVL	3EVL	4EVL	5EVL	1irAOCL	2irAOCL	3irAOCL	4irAOCL	5irAOCL	1EVR	2EVR	3EVR	4EVR	5EVR	1irAOCR	2irAOCR	3irAOCR	4irAOCR	5irAOCR
1EVL	-	n.s	n.s	n.s	n.s	n.s	n.s	n.s	n.s	n.s	n.s	n.s	n.s	n.s	n.s	n.s	n.s	n.s	*	*
2EVL		-	n.s	n.s	n.s	n.s	n.s	n.s	n.s	n.s	n.s	*	n.s	n.s	n.s	n.s	n.s	n.s	n.s	n.s
3EVL			-	n.s	n.s	n.s	n.s	n.s	n.s	n.s	n.s	n.s	n.s	n.s	n.s	n.s	n.s	n.s	n.s	n.s
4EVL				-	n.s	n.s	n.s	n.s	n.s	n.s	n.s	n.s	n.s	n.s	n.s	n.s	n.s	n.s	*	*
5EVL					-	n.s	n.s	n.s	n.s	n.s	n.s	n.s	n.s	n.s	n.s	n.s	n.s	n.s	n.s	n.s
1irAOCL						-	n.s	n.s	n.s	n.s	n.s	n.s	n.s	n.s	n.s	n.s	n.s	*	*	n.s
2irAOCL							-	n.s	n.s	n.s	n.s	n.s	n.s	n.s	n.s	n.s	n.s	n.s	n.s	n.s
3irAOCL								-	n.s	n.s	n.s	n.s	n.s	n.s	n.s	n.s	n.s	n.s	n.s	n.s
4irAOCL									-	n.s	n.s	n.s	n.s	n.s	n.s	n.s	n.s	n.s	n.s	n.s
5irAOCL										-	n.s	n.s	n.s	n.s	n.s	n.s	n.s	n.s	n.s	n.s
1EVR											-	n.s	n.s	n.s	n.s	n.s	n.s	n.s	n.s	n.s
2EVR												-	n.s	n.s	n.s	n.s	n.s	n.s	n.s	n.s
3EVR													-	n.s	n.s	n.s	n.s	n.s	n.s	n.s
4EVR														-	n.s	n.s	n.s	n.s	n.s	n.s
5EVR															-	n.s	n.s	n.s	n.s	n.s
1irAOCR																-	n.s	n.s	n.s	n.s
2irAOCR																	-	n.s	n.s	n.s
3irAOCR																		-	n.s	n.s
4irAOCR																			-	n.s
5irAOCR																				-

N= 6374 sequences for each samples, R, roots; L, leaves; * (0.01-0.05) marginally significant; n.s (> 0.1) not significant.

Table S5. Pairwise ANOSIM did not differ significatly among rosette (young) and elongated, flowering (old) deveoplmental stages.

Pairwise Tests		
Groups	R Statistic	Significance Level
EVR-young, EVR-old	0.917	0.1
EVR-young, irAOCR-young	0	1
EVR-young, irAOCR-old	1	0.1
EVR-young, EVL-young	0	0.667
EVR-young, EVL-old	1	0.1
EVR-young, irAOCL-young	1	0.333
EVR-young, irAOCL-old	1	0.1
EVR-old, irAOCR-young	1	0.1
EVR-old, irAOCR-old	0.704	0.1
EVR-old, EVL-young	0.5	0.1
EVR-old, EVL-old	0.852	0.1
EVR-old, irAOCL-young	1	0.1
EVR-old, irAOCL-old	0.963	0.1
irAOCR-young, irAOCR-old	1	0.1
irAOCR-young, EVL-young	0.5	0.333
irAOCR-young, EVL-old	1	0.1
irAOCR-young, irAOCL-young	1	0.333
irAOCR-young, irAOCL-old	1	0.1
irAOCR-old, EVL-young	0.417	0.1
irAOCR-old, EVL-old	0.926	0.1
irAOCR-old, irAOCL-young	0.833	0.1
irAOCR-old, irAOCL-old	0.852	0.1
EVL-young, EVL-old	0.083	0.4
EVL-young, irAOCL-young	0	1
EVL-young, irAOCL-old	0.083	0.4
EVL-old, irAOCL-young	-0.333	0.9
EVL-old, irAOCL-old	-0.148	0.9
irAOCL-young, irAOCL-old	-0.5	1
Global	0.619	0.001

Table S6. List of OTUs significantly different among EV and ir*AOC* leaves and roots at genera (or higher) level retrieved by pyrosequencing.

OTU numbers	Genus (or higher)	Leaves		Roots		ANOVA test	
		EVL	ir*AOCL*	EVR	ir*AOCR*	Communities (EVL*ir*AOCL**EVR* ir*AOCR*)	
		Average proportion %				$F_{3,16}$	*p*
Otu51	*Arthrobacter*	0^{A}	0^{A}	0.12^{B} (0.04)	0.15^{B} (0.06)	3.67	0.03
Otu92	*Phenylobacterium*	0^{A}	0^{A}	0.04^{B} (0.01)	0.04^{B} (0.01)	5.56	0.008
Otu123	*Nocardioides*	0^{A}	0^{A}	0.03^{B} (0.01)	0.04^{B} (0.01)	7.48	0.01
Otu27	*Rhizobium*	0^{A}	0^{A}	0.56^{B} (0.16)	0.39^{B} (0.2)	5.15	0.01
Otu60	*Cupriavidus*	0.01^{A} (0.009)	0.01^{A} (0.004)	0.09^{B} (0.01)	0.11^{B} (0.03)	7.24	0.0028
Otu63	*Enterobacter*	10.1^{C} (3)	7.2^{BC} (2.4)	2.65^{AB} (0.9)	0.9^{A} (0.3)	4.19	0.02
Otu28	*Methylophilaceae*	0.03^{A} (0.03)	0.03^{A} (0.03)	0.44^{B} (0.17)	0.71^{B} (0.2)	6.28	0.005
Otu285	*Pseudomonas*	0.03^{A} (0.01)	0.02^{A} (0.008)	0.15^{B} (0.05)	0.08^{AB} (0.01)	3.85	0.03
Otu56	*Agrobacterium*	0.05^{A} (0.03)	0.03^{A} (0.03)	0.14^{B} (0.04)	0.06^{AB} (0.02)	3.52	0.03
Otu0	*Serratia*	28^{B} (4)	14.23^{A} (4.3)	8.53^{A} (3)	6.86^{A} (2.1)	7.67	0.002
Otu13	*Mycoplana*	0.02^{A} (0.01)	0.02^{A} (0.02)	1.68^{B} (0.4)	1.8^{B} (0.8)	4.43	0.01
Otu24	*Achromobacter*	0.14^{A} (0.08)	0.01^{A} (0.01)	0.48^{AB} (0.16)	0.68^{B} (0.2)	3.64	0.03
Otu3	*Oxalobacteraceae*	1.69^{AB} (1.1)	0.34^{A} (0.1)	4.38^{BC} (0.9)	5.8^{C} (1.2)	6.58	0.005
Otu398	*Janthinobacterium*	0.01^{A} (0.004)	0.01^{A} (0.006)	0.26^{B} (0.05)	0.16^{B} (0.07)	7.08	0.003

R, roots; L, leaves; different letters indicates significant differences with Fisher's PLSD test; *P*<0.05.
